# New materials of the rare fossil mustelid *Cernictis hesperus* (Carnivora, Mammalia) from the Pinole Tuff genotype locality in California

**DOI:** 10.7717/peerj.20894

**Published:** 2026-05-15

**Authors:** Z. Jack Tseng

**Affiliations:** 1 Department of Integrative Biology, University of California, Berkeley, CA, United States of America; 2 Museum of Paleontology, University of California, Berkeley, CA, United States of America

**Keywords:** Fossils, Carnivora, Mammals, Biogeography, Dispersal

## Abstract

The Neogene fossil record of predatory mammals indicates periodic dispersals across the Beringian land bridge. Among the documented immigrant species in the weasel family (Mustelidae), *Cernictis hesperus* is among the most poorly known genotype species because of the highly fragmentary holotype. Here I report a new specimen representing the species from its type locality in the Pinole Tuff Formation, California. Associated left and right dentaries show that *C. hesperus* possesses a first premolar, unlike all other known species of the genus. Furthermore, incisor alveoli indicate the presence of a full incisor toothrow that is similarly crowded compared to the cheek teeth. These new morphological characteristics improve the diagnosis of the genus and species for the first time in nearly a century since the initial publication on the taxon.

## Introduction

Among the carnivoran mammals that made the cross-continental trek through the Beringian land bridge over the course of the past ∼20 million years, perhaps few are as poorly characterized in North America as the mustelid *Cernictis* (Qiu, 2003; Woodburne, 2004). Originally described as *Cernictis hesperus* from the late Miocene Pinole Tuff Formation in the San Francisco Bay Area (Hall, 1935) of North America, the genus was known for nearly eight decades only from a single fragmentary dentary preserving the fourth premolar and first molar. A second species was described by Baskin (2011) on the basis of a four-tooth jaw, and two more species were described by Jiangzuo et al. (2024) from multiple nearly complete dentaries, a maxillary fragment, and a cranium.

The paucity of morphological information in the genotype complicates not only the identification of additional material belonging to this genus, but also its phylogenetic affinities. *Cernictis* have been variably placed in the mustelid subfamily Galictinae (Baskin, 2011), tribe Lyncodontini in the subfamily Ictonychinae (Jiangzuo & Wang, 2023), or tribe Ictonychini in subfamily Ictonychinae (Jiangzuo et al., 2024). Although Jiangzuo et al. (2024) supported their higher level taxonomic assignment for *Cernictis* using total evidence analysis, they recognized that the posterior probability obtained for a tribe Ictonychini containing *Cernictis* is low (0.53). Therefore, there is no current consensus on the phylogenetic position of this genus.

In this study I report the first additional fossil material referrable to *C. hesperus* from the Pinole Tuff Formation in nearly a century, adding new dental morphological information to the poorly characterized genotype species and providing high-resolution 3D models to facilitate future work in resolving the classification and phylogenetic relationships of this fossil carnivoran species.

**Paleontological and geological setting**. The Pinole Tuff (*sensu lato*) contains tephra layers thought to be sourced from the Sonoma volcanic field to the north, as well as reworked volcaniclastic and non-volcanic sediments overlying the main tuff layers. In a recent study of the tephrochronology of the Mount Diablo region of the greater San Francisco Bay, Sarna-Wojcicki et al. (2021) restricted the name “Pinole Tuff Complex” to the basal tephra layers and renamed the sediments above the tephra layers to be part of the Contra Costa Formation. In 2023, I attempted to relocate the two vertebrate fossil localities discussed in this study, but extensive housing development, fencing, and landscaping in the general locality area made it impossible to track down precise stratigraphic levels of the locations where specimens were originally collected. Because of this uncertainty, my broad usage of the Pinole Tuff includes both the tephra layers as well as the reworked volcanoclastic sediments. Thus, the mean age of the Pinole Tuff Complex is used herein as the maximum age of the fossils. The overlying Roblar Tuff has been dated at 6.1–5.7 Ma (Sarna-Wojcicki et al., 2021) and represents an upper bound to the same fossils discussed below.

The type locality of *Cernictis*, Pinole Junction 1 (Locality -2572), is represented by 110 accessioned vertebrate fossil specimens in the University of California Museum of Paleontology (UCMP). The fauna is dominated in specimen quantity by the antilocaprid *Sphenophalos* and the equid *Pliohippus*, with a few specimens representing the antilocaprid *Capromeryx*, the ground sloth *Megalonyx,* and the borophagine canid *Borophagus* (Stirton, 1939). The spike-toothed salmon, *Oncorhynchus rastrosus*, is known from both the type locality and also Pinole Junction 2 (Sankey et al., 2016). The only described medium to small size mammal from Pinole Junction 1 had been the holotype of *Cernictis hesperus* (Hall, 1935). Pinole Junction 2 (Locality V3425), from which the referred specimen of *C. hesperus* described in this study was collected, is represented in the UCMP collection by 786 specimens. In addition to sharing the antilocaprid, equid, borophagine canid and salmonid taxa with Pinole Junction 1, this second locality also records the canid *Eucyon davisi* (Tedford, Wang & Taylor, 2009), the mustelid *Plesiogulo* (Harrison, 1981), the cricetid rodent *Miotomodon* (Korth, 2011), the presently described additional specimen of *Cernictis*, and several specimens representing undescribed small mammals and reptiles. Overall, the vertebrate community of the Pinole Tuff local fauna exhibits transitional characteristics between North American coastal range assemblages of Clarendonian North American Land Mammal Age (*e.g.*, Macdonald, 1948) and those of near-modern communities of Plio-Pleistocene age (Stirton, 1939).

## Materials and Methods

The genotype of *Cernictis hesperus*, UCMP 22968, was CT scanned using a GE Phoenix Nanotom M microCT system in the Functional Anatomy and Vertebrate Evolution Laboratory, University of California, Berkeley, at 15.03 micrometer voxel size with a voltage setting of 130 kV, current of 95 µA, and reconstructed from 1,500 raw projection images into a TIFF stack of 1,910 images. The referred specimen, UCMP 57666, was scanned using the same system at 25.05 micrometer voxel size with a voltage setting of 110 kV, current of 110 µA, and reconstructed from 1,500 projection images into a TIFF stack of 1,893 images. Both CT image stacks were imported into 3D Slicer software using the Slicermorph module, and automatically thresholded using the otsu algorithm implemented in the segment editor module (Fedorov et al., 2012; Rolfe et al., 2021). The resulting 3D meshes were exported in *.stl format and aligned in Geomagic Wrap 2020 (Hexagon AB, Stockholm, Sweden) to anatomical planes, and screenshots taken of the lateral, medial, and occlusal views to generate the specimen figure. All linear measurements of the UCMP specimens were taken on the digital meshes from within Geomagic Wrap to the nearest 0.01 mm. Measurement values for other *Cernictis* specimens were taken from Baskin (2011) and Jiangzuo et al. (2024). 3D meshes of the UCMP specimens are available for download on FigShare at DOI: 10.6084/m9.figshare.30445088 and 10.6084/m9.figshare.30570038.

## Results

### Systematic paleontology

**Table:** 

Order CARNIVORA Bowdich (1821)
Family MUSTELIDAE Batsch (1788)
Genus *CERNICTIS*Hall, 1935

**Type species**. *Cernictis hesperus* Hall, 1935.

**Type specimen**. UCMP 22968, left dentary fragment with p4-m1.

**Type locality**. Pinole Junction 1 (UCMP Locality -2572), Pinole Tuff Formation, Contra Costa County. Late Hemphillian North American Land Mammal Age (NALMA; ∼6.25 Ma based on Sarna-Wojcicki et al. (2021)).

**Included species**. *Cernictis hesperus* Hall, 1935 *, C. repenningi* Baskin, 2011*, C. baskini* Jiangzuo et al. (2024), *C. lufengensis* Jiangzuo et al. (2024).

**Emended diagnosis**. Three to four premolars, crowded and with little or no space between adjacent cheek teeth. When present, the p1 is single-rooted, p2-m1 two-rooted. The p4 has a posterior accessory cuspid (PAC), and the p3 may or may not have a PAC. The m1 talonid is less than half the length of the trigonid. The moderately developed m1 metaconid is the same height as or lower than the m1 paraconid. The m1 talonid basin is bound on the lingual and distal sides by a continuous cingulum, which borders the distal face of a well-developed hypoconid. Cranial characters as in Jiangzuo et al. (2024).

**Figure 1:**
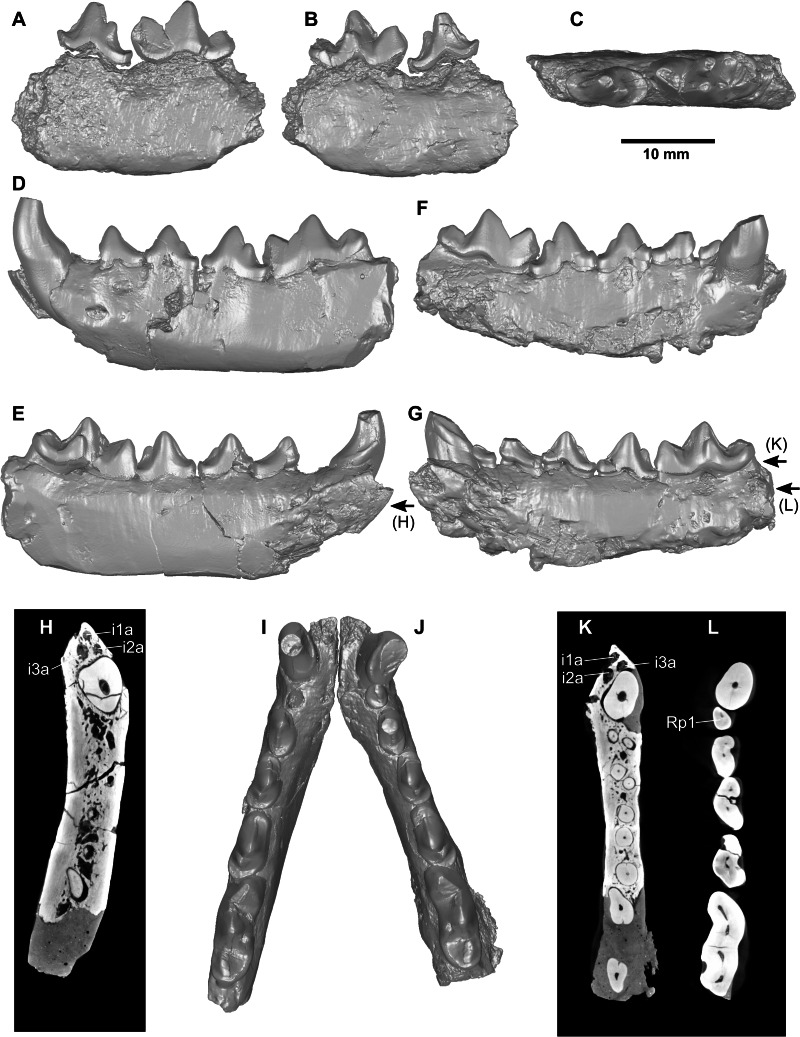
Specimens of *Cernictis hesperus*. Holotype specimen, UCMP 22968: (A) lateral view, (B) medial view. (C) occlusal view. Referred specimen, UCMP 57666: (D) lateral view of left dentary, (E) medial view of left dentary, (F) lateral view of right dentary, (G) medial view of right dentary, (H) transverse CT slice showing left incisor alveoli, (I) occlusal view of left dentary, (J) occlusal view of right dentary, (K) transverse CT slice showing right incisor alveoli, (L) transverse CT slice showing enamel-dentine junction of p1. Arrows with letters in parentheses show position of transverse CT slices. Abbreviations: *i1a*, first incisor alveolus; *i2a*, second incisor alveolus; *i3a*, third incisor alveolus; *Rp1*, right first lower premolar.

*Cernictis hesperus* (Fig. 1; Table 1)

**Diagnosis**. “Size…slightly greater than in *Plionictis parviloba* Cope; p4 long with accessory posterior cusp; m1 short, talonid less than half length of trigonid; basal cingulum extended up anterior border of paraconid, giving appearance of concavity to lateral face of paraconid-protoconid blade; metaconid well developed and connected with tip of protoconid by a distinct ridge; talonid semi-basined; base of metaconid continuous with lingual margin of talonid; base of protoconid separated from buccal margin of talonid by distinct notch; basin of talonid enclose by ridge apparently without well developed entoconic [sic] or hypoconid” (Hall, 1935).

**Emended Diagnosis**. Four premolars are present in some individuals; p3 with less developed posterior accessory cuspid compared to p4; p1 and p2 single-cusped with a long posterior cingulum; a single mental foramen at level of the interdental position between p1 and p2; canine with a broad basal lingual ridge that trends distal-ventrally and ends in a short posterior cingulum; other traits as in the diagnosis provided by Hall (1935).

**Differential Diagnosis**. Updating those provided in Jiangzuo et al. (2024): differs from *Baranogale* and *Vormela* in some individuals retaining p1; differs from *Martes* by a more robust m1 with more elongate protoconid. Other features as in Jiangzuo et al. (2024).

**Referred specimen**. UCMP 57666, mandibular fragment with left i1-i3 alveoli, c, p1 alveolus, and p2-m1, and right i1-i3 alveoli, c, p1-m1. Collected by D. Webb as part of UC Berkeley Paleo 254 course field trip in 1960.

**Locality**. Pinole Junction 2 (UCMP Locality V3425), Pinole Tuff Formation, Contra Costa County. The UCMP catalogue record for this locality states that the locality is “SE $ \frac{1}{4} $ of Sec. 16. T2N, R4W Mare Island Quadrangle. In middle of bank, 12 ft above Southern Pacific Railway track, approx. 504 yards west of Pinole Junction. The specimens were found in a layer of gravel and volcanic ash. The site is 10 ft west of the first outcrop of ash that occurs on the north side of the Right of way. Approximately 64 yards west of U.C. Locality 2572, at presumedly, the same level.” Given this description and proximity to Pinole Junction 1 (UCMP Locality -2572), the locality of the *Cernictis* genotype, I tentatively interpret a similar age of late Hemphillian NALMA (∼6.25 Ma) for Pinole Junction 2.

**Table 1:** Dental measurements of *Cernictis* species. Data for *C. repenningi* from Baskin (2011); data for *C. baskini* and *C. lufengensis* from Jiangzuo et al. (2024). Data for UCMP specimens are from this study.

Taxon	Specimen	Age (Ma)	p1L	p1W	p2L	p2W	p3L	p3W	p4L	p4W	m1L	m1W	m1tr
*Cernictis hesperus*	UCMP 22968	∼6.25							8.59	3.96	11.37	4.96	8.34
*Cernictis hesperus*	UCMP 57666 (L)	∼6.25			5.29	2.88	6.16	3.14	7.72	3.70	11.69	4.33	8.52
*Cernictis hesperus*	UCMP 57666 (R)	∼6.25	2.87	2.46	5.10	2.96	6.29	3.04	7.80	3.65	11.59	4.29	8.54
*Cernictis repenningi*	UALP 8079	6.5			4.70	2.60	5.40	3.40	7.30	4.20	10.60	5.10	7.50
*Cernictis baskini*	F:AM 22342	7.0–5.7			4.19	2.92	5.15	3.12	6.47	3.52	10.02	5.00	7.22
*Cernictis lufengensis*	IVPP V6885.1	7.0–4.9					3.64	2.12	4.62	2.64	7.24	3.04	4.52
*Cernictis lufengensis*	ZT2015-0321	6.5–6.0					5.10	2.40	5.88	2.84	8.27	3.74	5.67

**Notes.**

Abbreviations Ma mega-annum p premolar m molar L length W width tr carnassial trigonid

All measurements are reports in mm to the nearest 0.01 mm. For institutional abbreviations see text.

**Description**. The newly referred specimen is composed of two hemimandibular fragments belonging to the same individual. The ramus portion of the right fragment is incomplete, broken ventrally along the toothrow and completely missing ventral to the m1, and has been partially reconstructed using plaster. The left fragment is better preserved, with intact ramus from the canine to the position of the m1 protoconid; there is minor reconstruction of the ramus posterior to the m1 protoconid in plaster material. The partially preserved mandibular symphyses are moderately rugose, and articulate closely with each other when placed in anatomical position.

The incisor alveoli are preserved on both sides of the jaw, but no sign of incisors crowns or roots are present (suggesting that the incisors were lost prior to permineralization). Cross-section images from microCT scans of the specimen clearly show the presence of three incisor alveoli. The alveoli are staggered and crowded in a narrow portion of the mandibular ramus between the mandibular symphysis and the canine (Figs. 1H–1L). I identified the alveolus closest to the canine as i3 alveolus, the posteriorly offset opening as i2 alveolus, and the antero-medial alveolus as the i1 alveolus. The canine teeth are broken roughly eight mm above the bases of the crowns, which has a medial cingulum that runs from the distal base of the tooth and gradually blends into a vertical ridge lining the medial face of the tooth.

Perhaps the most pronounced morphological trait that the new specimen adds to the concept of the species and genus is the confirmed presence of p1. A single-rooted, single-cusped p1 is present on the right side, closely appressed to the distal face of the canine tooth. The tooth is missing on the left side, but a well-preserved alveolus between the canine and p2 signifies its previous presence. The p2 is preserved on both sides of the mandible, and shows a two-rooted, single-cusped tooth that is similar to p1 in shape but much larger in size. The p3 has a centrally positioned main cuspid, and a small posterior accessory cuspid that is halfway between the tip of the main cuspid and a moderately developed posterior cingulum. The p4 is similar in overall morphology to the p3, but with a relatively larger posterior accessory cuspid, and a small anterior cingulum.

The m1 tooth is preserved on both sides of the mandible. The protoconid is the tallest cuspid on the tooth, and the paraconid and metaconid are similar in height. The talonid has a strong lingual cingulum that runs from the distal base of the protoconid along the lingual and distal sides of the basin, ending just around the labial side a well-developed hypoconid. The labial contact between the protoconid and hypoconid is in the form of a trenchant notch.

**Comparison.** Relative to the p4 and m1 on the genotype specimen (UCMP 22968), the referred specimen has a relatively shorter p4 and longer m1. The shorter p4 on the referred specimen makes them appear more robust than that on the holotype; the longer and narrower m1 on the referred specimen makes them appear more slender than on the holotype. Both of these differences are minor and considered here to be intraspecific variation. As in all other described species of *Cernictis*, the m1 talonid is less than half the length of the m1 trigonid (Table 1). Unlike any of the other *Cernictis* specimens (including the holotype), the referred specimen clearly shows the presence of p1 in an otherwise similarly crowded cheek dentition as in other *Cernictis* specimens. Comparisons of the premolar dimensions among species of *Cernictis* reinforce the observation by previous authors that *C. hesperus* is the largest species within the genus, followed by *C. repenningi,* then *C. baskini*, and finally, *C. lufengensis* (Table 1).

The uncertainty of the phylogenetic position of *Cernictis* (Baskin, 2011; Jiangzuo & Wang, 2023; Jiangzuo et al., 2024) makes it difficult to identify other closely related genera against which *Cernictis* should be compared. In an attempt to better contextualize the new information represented by the referred specimen of *Cernictis hesperus* described in this study, here I compare the species with regard to other taxa to which previous authors have compared. Hall (1935) suggested similarity between *C. hesperus* and the living mustelid genera *Martes* and *Gulo* but did not provide justification for it. In overall dental morphology, the lower dentition in *C. hesperus* is morphologically similar to those of *Martes* in the presence of p1, the absence of posterior accessory cuspids in p2-p3, and a well-developed posterior accessory cuspid on p4. However, the m1 protoconid in *C. hesperus* constitutes a larger proportion of total tooth length than in *Martes*. Jiangzuo & Wang (2023) noted that *Cernictis* has a notably more trenchant dentition compared to contemporaneous *Lutravus*; this difference seems to hold in the newly referred material described herein, with the modification that both *Lutravus* and *Cernictis* are now known to possess p1 at least in some species. Conversely, previous comparisons using the loss of p1 in *Cernictis* as one of the characteristics separating it from *Trochictis, Sinictis, Circamustela,* and *Aragonictis* should be discarded (Baskin, 2011; Jiangzuo et al., 2024).

## Discussion

The original description of *Cernictis hesperus* by Hall, 1935 was based on a highly fragmentary left dentary with p4-m1. This report significantly improves the representation of the species from its genotype locality area by adding descriptions of the canines, all premolars, and incisor alveoli. The main amendment to the diagnosis of the genus is the variable presence of p1 (rather than absence of p1) (Baskin, 2011; Jiangzuo et al., 2024). The presence of p1 is typically considered to be a plesiomorphic state of unreduced premolar dentition in mustelids, suggesting that *C. hesperus* may represent an earlier diverging member of the genus. This interpretation is consistent with the phylogenetic framework proposed by Jiangzuo et al. (2024), which places the two North American species (*C. hesperus* and *C. repenningi*) as earlier diverging than the east Asian species *C. lufengensis* and *C. baskini*. Chronologically, all known *Cernictis* specimens are near the late Miocene-Pliocene boundary (5–7 Ma; Table 1), and a more precise dating of both Asian and North American specimens is necessary to determine whether the genus dispersed to North America from Asia (Jiangzuo et al., 2024), or whether a rarer westward dispersal of *C. baskini* and *C. lufengensis* from the earlier diverging *C. hesperus* should be considered. At the very least, the genotype material for *Cernictis* is no longer the sole basis for future comparative analysis of the genotype species; associated left and right dentaries reported herein add substantial new morphological information to the understanding of this rare mustelid taxon.

## Conclusions

This study reports a new fossil specimen of the extinct mustelid *Cernictis hesperus*, providing additional morphological information to better characterize the taxon. The continued lack of cranial and other skeletal material of this species, as well as uncertainty in the stratigraphic distribution of the species at its genotype locality, both contribute to challenges associated with placing the taxon in a more robust phylogenetic framework. Additional discoveries are necessary to resolve these issues.

## Institutional abbreviations

F:AM: Frick Fossil Mammal Collection at the American Museum of Natural History, New York, USA
IVPP: Institute of Vertebrate Paleontology and Paleoanthropology, Chinese Academy of Science, Beijing, China
ZT: Zhaotong Institute of Cultural Relics Protection and Archaeology, Zhaotong, China
UALP: Desert Laboratory on Tumamoc Hill, The University of Arizona, Tucson, USA
UCMP: University of California Museum of Paleontology, University of California, Berkeley, USA.

## Additional Information and Declarations

### Data Availability

The following information was supplied regarding data availability: The UCMP 22968 and UCMP 57666 models are also available at MorphoSource: – https://doi.org/10.17602/M2/M793950. – https://doi.org/10.17602/M2/M809708. – https://doi.org/10.17602/M2/M794018.
